# Validity and Reliability of a Questionnaire on the Knowledge, Attitudes, Perceptions and Practices toward Food Poisoning among Malaysian Secondary School Students: A Pilot Study

**DOI:** 10.3390/healthcare11060853

**Published:** 2023-03-14

**Authors:** Pawitra Ramu, Malina Osman, Noor Azira Abdul Mutalib, Musheer A. Aljaberi, Kuo-Hsin Lee, Chung-Ying Lin, Rukman Awang Hamat

**Affiliations:** 1 Department of Medical Microbiology, Faculty of Medicine and Health Sciences, Universiti Putra Malaysia, Serdang 43400, Selangor, Malaysia; 2 Department of Community Medicine, Faculty of Medicine and Health Sciences, Universiti Putra Malaysia, Serdang 43400, Selangor, Malaysia; 3 Department of Food Service and Management, Faculty of Food Science and Technology, Universiti Putra Malaysia, Serdang 43400, Selangor, Malaysia; 4 Faculty of Medicine and Health Sciences, Taiz University, Taiz 6803, Yemen; 5 Department of Emergency Medicine, E-Da Hospital, I-Shou University, Kaohsiung 824, Taiwan; 6 School of Medicine, College of Medicine, I-Shou University, Kaohsiung 824, Taiwan; 7 Department of Emergency Medicine, E-Da Dachang Hospital, I-Shou University, Kaohsiung 807, Taiwan; 8 Institute of Allied Health Sciences, College of Medicine, National Cheng Kung University, Tainan 701401, Taiwan

**Keywords:** food poisoning, unhygienic food, eating behavior, validity, reliability, school, health promotion

## Abstract

Children in school settings are at risk of contracting food poisoning due to inadequate food safety practices and safe eating behaviors. This research aimed to develop a valid and reliable questionnaire on the knowledge, attitudes, perceptions, and practices (KAP^2^) toward food poisoning and its prevention among secondary school students. The questionnaire was developed by considering the Health Belief Model (HBM). A pilot study using a cross-sectional survey was conducted in Tangkak, Johor, among 30 selected students using a convenience sampling method. A pre-test was conducted on 15 secondary school students aged 13–17 years old prior to the pilot study, and they were excluded from the pilot study. The constructed knowledge was assessed using the difficulty and discrimination indices. Meanwhile, the reliability of the attitude, practice, and perception components in the questionnaire were assessed using Cronbach’s alpha. Regarding knowledge (34 items), the difficulty index showed that most items (*n* = 28) were easy, while one was difficult, and the remaining five were within an acceptable range. In addition, the discrimination index of the knowledge component (34 items) showed that twenty-two, three, and one had good, acceptable, and excellent ranges, respectively. Only eight items had a low discrimination power. All items of the attitude and practice components (10 items for each) showed a corrected item-total correlation value of >0.30. Only four out of twenty-one items of the perception component showed an unacceptable range of <0.30. However, following a discussion with the experts, all items were retained. With the incorporation of the HBM, the 75-item adapted food poisoning KAP^2^ questionnaire is valid and reliable. It can be utilized to measure and generate food poisoning KAP^2^ among secondary school students in Malaysia.

## 1. Introduction

Unhealthy eating behavior has contributed to the development of many chronic non-communicable diseases (NCDs) with significant mortality and morbidity worldwide [1]. This is further complicated by the occurrence or recurrence of foodborne infectious diseases including food poisoning, which is also associated with this problematic eating behavior [2]. Food poisoning or food-borne disease is a major public health concern worldwide. According to the World Health Organization, there are about 600 million cases of food-borne diseases annually, which is equivalent to 1 in 10 people around the world falling sick to food poisoning each year. Moreover, of 420,000 people who succumb to food-borne diseases, 125,000 (30%) of them are children less than five years old [3]. In Malaysia, there was an annual incidence of 50.90 per 100,000 population of food poisoning with a mortality rate of 0.03 per 100,000 population in 2019 [4,5,6]. Food poisoning cases in schools are now in an alarming state. Hyun-Dong et al. [7] demonstrated that a 24% attack rate was reported by a South Korean girls’ high school in 2008, while a 27.3% attack rate was reported by a South Korean high school in 2019 [8]. More recently, a large-scale food poisoning outbreak has been reported in Japan involving 3000 elementary and junior high school students [9]. In addition, 32 out of 202 episodes (15.8%) of food poisoning were reported in Ministry of Education (MOE) schools, while 17 episodes (8.4%) occurred in schools that were not registered with the MOE in Malaysia [10]. One state in Malaysia (Terengganu) also recorded 23 out of 33 (63.6%) episodes of food poisoning outbreaks among school children. Among 21 outbreak cases, 20 MOE schools (95.3%) and one (4.75%) non-MOE school were involved. In addition, food poisoning outbreaks were reported to be higher among students in secondary schools (13–17 years old) compared to students in primary schools (7–12 years old) (81% versus 19%), while twelve (57.1%) and nine (42.9%) outbreaks occurred in schools located in urban and rural areas, respectively [11].

The causes for the outbreaks of food poisoning in schools are multifactorial [12]. Improper food preparation and storage and poor personal hygiene are among the main contributing factors [13]. Moreover, the infectious agents of food poisoning might originate from humans, animals, or animal products [14,15]. Considering children and young adolescents are more likely to be involved in risky or unhygienic food handling practices and would exclusively spend their mealtime in school canteens, the risk of food poisoning is extremely high [16,17]. Schools are recognized as crucial places for establishing health promotion and nurturing health-related behavior [18,19]. Once the appropriate behaviors are manifested in adolescence, these behaviors tend to be retained in adulthood [20]. Hence, educating consumers—in this case, school children—on safe food handling practices is pivotal to improve the risky eating behavior among them and further prevent or reduce the incidence rate of food-borne illnesses [21].

Tackling the problematic and unhygienic food handling behaviors among school children requires appropriate instructional strategies as their ages and levels of maturity are different; hence, their understanding of food safety may not be sufficiently acknowledged if a general approach is used [22]. Substantial studies have indicated that children and young adolescents must acquire adequate learning skills related to food safety as their levels of awareness, knowledge, and perception are still lacking [23,24,25,26]. Education should be provided to increase the knowledge of food safety and food-borne illnesses as it will substantially impact their future behavior toward food safety [16,27,28]. Hence, studies regarding knowledge, attitudes, practices, and perceptions (KAP^2^) using a valid and reliable instrument are critical in the prevention of food poisoning among students. Earlier investigations on KAP studies did not employ a validated questionnaire for children or young adolescents in school settings and were focused on the dining out behaviors among consumers [29,30,31,32]. It is critical to employ an accurate and trustworthy questionnaire to determine changes in respondents’ knowledge and behavior [33].

To our knowledge, data on KAP studies using validity and reliability assessment tools for preventing food poisoning among students are lacking. The current study aimed to create a valid and reliable KAP survey on food poisoning and its prevention among secondary school children while eating their meals at school compounds.

## 2. Materials and Methods

### 2.1. Study Design and Instruments

This cross-sectional study was conducted in the educational district of Tangkak in Johor, Malaysia, from May to September 2021, with the goal of validating the KAP questionnaire. This pilot study included 30 respondents based on the inclusion and exclusion criteria. The inclusion criteria were students aged 13–17 years old who were able to read and understand Bahasa Malaysia. Students who were absent during the data collection and did not obtain approval from their parent/guardian were excluded from this study.

A self-administered questionnaire was used to examine respondents’ knowledge, attitudes, practices, and perceptions toward food poisoning and its prevention impact. The Malay language was utilized in the questionnaire since it is Malaysia’s most widely spoken language [34]. A pre-test on 15 secondary school students was undertaken for the language clarity in Phase 1 of the questionnaire development process. Eventually, the improved questionnaire was applied to the pilot study in Phase 2. All parents of the respondents signed a written informed consent form. While obtaining the information, approximately five minutes was spent discussing the study’s goal and outcome with the respondents. The questionnaire took 15 to 30 min to complete.

### 2.2. Questionnaire Development

The questionnaire was constructed with five domains: sociodemographic profile, knowledge, attitude, preventive practice, and behavioral perceptions. Each of the questionnaire domains was elaborated as follows.

#### 2.2.1. Sociodemographic Profile

This section contained questions that were designed to learn more about the demographic characteristics of the respondents, such as their gender, age, ethnicity, and household income. Other ethnicities included in this section were indigenous peoples, Sabahan, and Sarawakian. The household income range was based on the median monthly household income of the Malaysian population, which was divided into three categories: the bottom 40% of low-income earners (B40), the middle 40% of average-income earners (M40), and the top 20% of top-income earners (T20), with median monthly incomes of MYR 3000.00, MYR 6275.00, and MYR 13,148.00, respectively [35].

#### 2.2.2. Knowledge toward Prevention of Food Poisoning

Respondents’ knowledge of food poisoning was assessed based on food poisoning agents, high-risk foods, signs and symptoms, complications, and prevention aspects [33]. In total, we included 34 items for the knowledge component. The responses were documented in the form of “Yes” and “No”. One mark was allocated to correct answers and zero marks were allocated to incorrect answers [36]. All scores were summed to obtain the summary score. The total score of the knowledge component ranged from 0% to 100%.

#### 2.2.3. Attitude toward Prevention of Food Poisoning

Attitude is the preferred method of acting in various situations to maintain a system’s beliefs and ideals. The application of the attitude component in a KAP study will usually be blended and permuted with other factors such as cognitive (thinking), affective (feeling), and behavioral (activity) factors. The attitude of respondents toward food poisoning prevention, treatment, and risk-related behaviors was measured using these subdomains according to the concept that was developed by Zainuddin et al. [36]. A total of 10 items were included in this part. All responses were scored on a 5-point Likert scale (1 = strongly disagree; 2 = disagree; 3 = uncertain; 4 = agree; 5 = strongly agree). Negative items were reverse-scored, and the results for each domain were added together to generate an overall attitude score. The attitude score ranged from 10 to 50.

#### 2.2.4. Practice toward Prevention of Food Poisoning

In the present study, the questions on self-reported food poisoning prevention practices were modified with adjustments from earlier studies [36,37]. Closed-ended questions were used to evaluate the food poisoning prevention technique. To check the consistency of the responses in answering the questions, the questions comprised both negative and positive remarks [38]. In this part, 10 items were included. A 5-point Likert scale was used, with the options “1 = never”, “2 = seldom”, “3 = sometime”, “4 = frequent”, and “5 = always”. All scores were added together, and negative responses were reverse-scored. The practice score ranged from 10 to 50.

#### 2.2.5. Perception toward Prevention of Food Poisoning

Respondents’ perceptions of food poisoning were constructed using Health Belief Model (HBM) structures. The HBM is one of the most important behavioral theories, and suggests that an individual is more likely to change his or her behavior and adhere to the guideline or module because appropriate constructs are designed based on their various perceptions and motivations [39,40,41]. Moreover, as the model suggested, health-related behavior is indirectly affected by perception, which can be influenced by knowledge and diverse demographic factors [41,42]. In the present study, six constructs of the HBM were included as follows: (i) risk of acquiring the disease (perceived susceptibility); (ii) bad outcome from the disease (perceived severity); (iii) future health behavior to be both valuable and practical (perceived benefit); (iv) minor obstacles to adopting the behavior (perceived barrier); (v) having the skills to utilize and perform a specific proposed behavior (self-efficacy); and (vi) having cues to stimulate their action (cues to action). The HBM highlights that high perceived risks, low perceived barriers, and high perceived benefits to action would increase the likelihood of an individual participating in the recommended behavior [40]. The HBM is a commonly utilized theoretical tool for analyzing food safety operations [43].

Perceived barriers (three items), benefits (three items), susceptibility (three items), severity (three items), self-efficacy (three items), and cues to action (three items) were developed as constructs in the present study. Three items for the health-seeking behavior construct were also included. Health-seeking requires an individual to understand the reasons that could motivate him or her to try to seek healthcare. This behavior can modulate the behavioral practices that are important in the development of health promotion programs or interventions [40]. In total, 21 items were included in this section. The questions on perceptions of food poisoning prevention in the current study were adapted with modifications from previous studies [19,43,44,45]. All constructs were rated on a 5-point Likert scale (1 = strongly disagree; 2 = disagree; 3 = uncertain; 4 = agree; 5 = strongly agree). All points were summed up, and negative items were reverse-scored. The perception score ranged from 21 to 105.

### 2.3. Content Validity

Content validity is the process of evaluating a new survey instrument to verify that it has the necessary questions while excluding those that are unimportant to a specific construct area [41,46,47]. In the present study, the validity of each construct in the questionnaire was assessed by seven experts comprising a science teacher, a school counsellor, and experts in the food safety, food microbiology, public health, medical microbiology, and community health fields. The questionnaire was created to cover the following components: (i) the items reflecting the theoretical framework employed; (ii) the items well-suited to the construct being assessed; and (iii) the items that suitably covered the major study goals.

### 2.4. Face Validity

Pre-testing is a critical step in detecting problems with items in a questionnaire, reducing respondent burden, determining accurate comprehension of the topic by the respondents, and ensuring that the arrangement of the questions does not influence how the respondents react. According to the suggestions and recommendations, an appropriate change can be made [41]. To ensure uniformity in language, the questionnaire was pre-tested on 15 secondary school students aged between 13–17 as shown in Table 1, and they were excluded from the 30 students who comprised the sample in the present pilot study. During the process, some terminologies were simplified (e.g., “pesticide residue” and “cross-contamination”). Once again, the updated items were pre-tested on the same respondents to verify that they understood the new phrasing. The pre-test was conducted a week prior to the validity test and a month prior to the pilot test. It is important to note that the pilot test did not include respondents who took part in the pre-test in the present study.

### 2.5. Data Analytic Plan

SPSS Version 22 statistical software (SPSS Inc., Chicago, IL, USA) was used to analyze the data, validate the questionnaire, and ensure its reliability. For categorical data, descriptive statistics were used to analyze the data, which were then displayed as the frequency and percentage.

#### Validity and Reliability Test

To determine the relevance, substance, language clarity, response time, and simplicity of understanding of a questionnaire, a validity test is recommended [34,41,48,49]. In the present study, the constructed knowledge was determined by analyzing the difficulty and discrimination indices [33,50,51]. The following formula was used to determine the difficulty index: (the number of accurate answers to the knowledge questions)/(the total number of responses, including both correct and incorrect answers) [52].

The discrimination index is a value that varies from −1.00 to +1.00 [53]. Discrimination indices below 0.20, between 0.20 and 0.24, between 0.25 and 0.35, and greater than 0.35 are considered low, acceptable, good, and excellent, respectively [54]. If the index has a negative value up to 0.19, the question requires some modifications and is considered a low-discrimination question. A highly discriminating item means that the item is properly answered by students with high test scores, but incorrectly answered by students with low test scores [55].

The discrimination index was calculated using the following formula: [(the number of respondents properly answering in the top 27% of the higher group)−(the number of respondents correctly answering in the bottom 27% of the lower group)]/(the number of respondents in the upper or lower group) [33,52,56]. The top 27% of respondents are classified as the upper group, with high grades, and the bottom 27% are classified as the lower group, with lower grades [33].

With regard to the difficulty index, a question is rated easy if the difficulty index is greater than 0.7, acceptable if it is between 0.3 and 0.7, and tough if it is less than 0.3 [54,57,58]. The higher the value of the difficulty index, the simpler the question [59].

The upper and lower 27% rule is extensively used in item analysis based on Kelly’s (1939) derivation. The difference between the correct answers can be calculated as a percentage of the higher 27% and lower 27% of the overall category to determine whether a question discriminates against high- and low-scoring test takers. Furthermore, most prior research considered 27% to be a perfect balance between the upper and lower groups when calculating their discrimination indices [11,33,52]. As a result, in the present study, 27% of respondents in the higher and lower categories who answered properly were included in the computation of the discrimination index.

Reliability is also an important feature of questionnaires used in public health and social science studies [60,61]. After examining the content validity and face validity, attitudes, practices, and perceptions were all examined for their internal consistency (or internal reliability). Cronbach’s alpha, inter-item correlation (0.30), item-total correlation (0.30), and Cronbach’s alpha if item deleted are four essential metrics used to verify this concept, according to the previous literature [62,63,64]. The utilized metrics can aid the researcher in deciding whether the items in the same construct are coherent with each other [65].

As for the reliability, a Cronbach alpha value of less than 0.5 was interpreted as unacceptable, while a value of 0.6–0.7 was interpreted as acceptable, and, finally, a value of 0.8 or greater was interpreted as an excellent level of reliability [61,66,67].

## 3. Results

### 3.1. Sociodemographic Factors

The frequency distribution of sociodemographic factors among the 30 respondents included in the pilot study is shown in Table 1. The majority (70%) of the respondents were female, and the remaining respondents were male (30%). The respondents were equally distributed between 13 years old and 17 years old. Regarding ethnicity, the respondents were predominantly Malay (60%), followed by Chinese and Indian (20% for each). Approximately 70% of the respondents were from families in the B40 category. About 30% of the respondents were from families in the M40 category, while none of the respondents had a T20 family background.

### 3.2. Knowledge toward Prevention of Food Poisoning

The difficulty index showed that out of 34 items, twenty-eight were too easy, one was difficult, and the remaining five were within an acceptable range. The difficulty index and discrimination index for each item of the knowledge construct of food poisoning are presented in Table 2. Out of the 34 items of the discrimination index, eight items had a low discrimination power, three items showed acceptable discrimination, twenty-two items showed good discrimination, and one item showed excellent discrimination. Hence, the selected items of the knowledge construct were discussed and amended accordingly.

### 3.3. Attitude toward Prevention of Food Poisoning

All 10 items in this section showed a corrected item-total correlation value of >0.30. Thus, none of the items were removed from the attitude toward prevention of food poisoning construct. The overall Cronbach alpha value was 0.941, indicating an excellent reliability level. Table 3 shows the corrected item-total correlation and Cronbach alpha if item deleted values.

### 3.4. Practice toward Prevention of Food Poisoning

Table 4 shows the item analysis of practice toward prevention of food poisoning. Ten items demonstrated a corrected item-total correlation value of >0.30. These items were retained in this section. All items showed an excellent level of reliability based on the overall Cronbach alpha value (0.855).

### 3.5. Perception toward Prevention of Food Poisoning

Table 5 shows the result of the construct validity of the perception toward prevention of food poisoning construct. Four items (items 9, 14, 16, and 18) showed a corrected item-total correlation value of <0.30. The items were as follows: “Pse3-Consuming unsafe food can make people fall sick”, “Pba2-Informing teachers about unhygienic activity in canteen would require more effort which is inconvenient”, “Cta1-My teacher gives a talk about food poisoning”, and “Cta3-My teacher constantly reminds me to practice hygienic activity”. When each item was deleted, the Cronbach alpha value was slightly increased to more than 0.770. However, these items were retained and amended based on the agreement of the research team as they are vital to measure the perception of students. The reliability of the items was acceptable as the overall Cronbach alpha value was 0.770.

## 4. Discussion

There have been several KAP studies on food safety and cleanliness among food handlers and consumers [31,32]. A study by Zaujan and colleagues [31] only focused on the development of a questionnaire about food poisoning and its prevention while dining out among consumers. Although students can be considered consumers, the questions designed for food poisoning in the context of teacher–student interaction and their perception were not sufficiently covered and properly validated in their study. In addition, only four out of six elements of the Health Belief Model (perceived barrier, perceived susceptibility, perceived severity, and perceived benefit) were included in their validated questionnaire, and not all of the items were suitable for school children [31].

Episodes of food poisoning in schools are associated with many factors [12]. Students typically develop food poisoning from eating food that is prepared in school canteens, dorm kitchens, or under supplemental food programs. Like general consumers, they always assume that food business operators are more accountable for food safety than themselves [68]. In addition, poor food safety practices, poor knowledge, and lack of concern about food safety among young consumers have been reported by several studies [68,69]. Most schools in Malaysia provide or prepare food for school children, but the students can also bring food from home or other food providers. Adequate knowledge of food safety is very important for students because they are also considered consumers. It has been documented that food poisoning can be reduced or prevented if school children have adequate knowledge of food safety and proper personal hygiene practices [70]. With continuous educational efforts, students can be empowered to fulfil their food safety role. For example, with adequate knowledge, they can recognize unhygienic activities practiced by food handlers in the school canteen and report them to the teachers.

Based on evidence from the literature, the purpose of the present study was to extend the knowledge of food poisoning by developing a valid and reliable KAP questionnaire on food poisoning and its prevention specifically targeting secondary school students. This study used the difficulty and discrimination indices to evaluate the knowledge components. The validation of the knowledge components was performed by calculating these indices. Quaigrain and Arhin [50] supported this idea as the difficulty and discrimination indices should be used to determine whether or not the concept is properly developed [50]. These indices can assist researchers in determining which elements are acceptable and which ones should be eliminated, improved, or kept. The item difficulty was utilized to distinguish hard questions from easy ones [52]. The discrimination index measures how well an instrument distinguishes between respondents who receive high and low scores, as well as between those who are informed and those who are not [51]. Meanwhile, in the present study, the selected items based on the discrimination and difficulty indices were discussed among the researchers, and a few amendments were made. The amendments were related to the structure of the sentences and the usage of words that tend to confuse respondents. To avoid the loss of crucial items, it is vital to classify the suitable items for the research and to interpret the data with prudence. According to Koo et al. [52], a discrimination index of below 0.2 should be excluded. Low and negative discrimination indices are frequently caused by incorrect keying, inappropriate question wording, or poor respondent preparation before answering questions [59]. In the present study, eight questions had low discrimination power. These questions were considered easy but could not differentiate between lower and higher achievers among the students. It has been demonstrated that items that appraise the evaluation and explanation domains are more likely to have good discrimination power [71]. However, items that appraise the remembering and understanding levels are expected to have low discrimination power [72]. According to Mehrens and Irvin [73], one of the reasons for low discrimination power is the existence of easy items. However, we need such items to have an adequate and representative sample of the content and objectives. For example, food poisoning is associated with food that is contaminated with viruses and parasites. Indeed, these causative agents are responsible for a significant number of food poisoning outbreaks [74]. The students must fully grasp this fundamental concept in order to comprehend the causes of food poisoning. Hence, these items were kept in the present study despite having low discrimination power. Furthermore, the item’s purpose in relation to the total measurement may influence the magnitude of its discriminating power [50,73].

It is worth noting that the higher the level of discrimination, the better the item. However, removing these low-discriminating items could seriously impair test validity [50]. In this case, we might expect to find that “low positive indices of discrimination are the rule rather than the exception” [50,75]. Moreover, these items might be helpful in identifying students who lack a basic understanding of food poisoning. Nevertheless, these items could still be useful if the target students already have a certain level of food poisoning knowledge. Therefore, we recommend future research to take into account the study population when determining whether to include or delete these eight items or to completely revise them [50,76].

In the present study, all questions were designed to explore the basic understanding of food poisoning among secondary school students. Hence, it is expected that they need to remember and understand the topic before attempting to answer the questions. These questions were not only discriminatory but also adequately understood by all students; hence, the students correctly responded to each item in the knowledge component. Well-designed questions are known to create new insights and could promote broad exploration of the subject matter [77]. On the other hand, poorly constructed questions would lead to learning constraints and confusion among the students [78].

Internal consistency was assessed for the attitudes, practices, and perceptions among the secondary school children in the present study. Items that measure the same specific construct were assessed via corrected item-total correlations [65]. Our findings showed that the well-correlated items within the construct scope can be evaluated using the value of the inter-item correlation. The homogeneity of the items in the construct was established by assessing the item-total and inter-item correlations [79].

In the present study, all items in the constructs had inter-item and item-total correlation values of >0.3, except for the four items in the perception component. These four items were distributed in the three subdomains of perception as follows: perceived severity (one item), perceived barrier (one item), and cues to action (two items). When these items were deleted, there was a slight increase in the Cronbach alpha values for this construct. For example, “PSe3-Consuming unsafe food can make people fall sick” was the subdomain of perceived severity, and when the item was deleted, the Cronbach alpha value increased to 0.779 from 0.770. However, these items were amended and retained following the extensive discussion among our experts in the present study. Expert opinion can be used to create the necessary information for prior construction, especially when there is insufficient information in the literature [80]. In addition, collective data obtained from these items are useful for the development of health education modules on food poisoning among students. The term “unsafe food” is used to represent food that is contaminated with infectious agents as the cause of food poisoning. People who consume “unsafe food” would eventually fall sick. This term has been commonly used in several surveys on food safety [19,81]. In addition, the concepts of safe and unsafe foods are among the major key issues raised by the authorities pertaining to food safety [82]. Perceived personal threat and coping abilities are the central constructs of protection motivation theory [83]. A study among young adolescents on the frequency of brushing their teeth indicated that perceived severity had a preventive influence on the risk of brushing teeth less than twice daily [84]. The assessment of perceived severity is vital to evaluate how one perceives an illness (food poisoning in this case), which can influence their emotional response to the illness and their coping behaviors toward treatment [85,86]. Thus, it is important to assess students’ readiness concerning their risk of acquiring food poisoning when unsafe food is consumed. This readiness can be improved by increasing their knowledge and engagement through educational activities, including role-play that mimics real-life situations and gamification [87,88].

Similarly, for “PBa2-Informing teacher about unhygienic activity in a canteen would require more effort which is inconvenient (perceived barrier)”, a minute increase in the Cronbach alpha value was found when it was deleted, increasing to 0.773 from 0.770. Regarding the perceived barrier subdomain, it is very important to explore the potential communication gap between students and their teachers about reporting unhygienic activities in the school canteen. Thus, existing guidelines can be modified, or new interventions can be designed by including effective healthcare promotion and communication strategies, and finally can be implemented to reduce the burden of food poisoning among students. For example, the school administrative department can provide a platform for the students to give their feedback about hand hygiene and food handling practices in the canteen or the canteen’s unhygienic environment using a newly developed web-based application system such as FOODA lyzer© [89]. Effective communication skills are vital in influencing people to make healthy decisions and promoting healthy behaviors [90]. Patients’ anxiety, heavy workload, pain, and physical discomfort were identified as the main perceived barriers to therapeutic communication among nurses and patients in Sri Lanka [91]. Apart from that, an appropriate channel of communication can be provided to limit the barrier between teachers and students, or the students themselves can be trained to be “health inspectors” in schools.

Regarding the cues to action subdomain, items such as “Cta1-My teacher gives a talk about food poisoning” and “Cta3-My teacher constantly reminds me to practice hygienic activity” were also retained in the present study. Since food poisoning outbreaks are common in schools, and since cues to action are commonly associated with social influence or any events that could trigger one to change or adapt their behavior, these items are very important [92]. The behaviors of teachers, such as caring actions and praise, could inspire students to change their negative behaviors [93]. Continuous teacher reminders could motivate students to adapt or change their preventive behavior toward food poisoning. Students not only need to be told why they should practice good preventive behaviors, but they also need to be reminded and encouraged to carry out such behaviors. It has been shown that both cues to action and self-efficacy could improve nutritional behavior among Iranian adolescents [94].

The internal consistencies of the items that were developed for the constructs of attitudes, practices, and perceptions were examined based on the Cronbach alpha values. The overall Cronbach alpha values in the present study were 0.941, 0.855, and 0.777 for attitudes, practices, and perceptions, respectively. Cronbach’s alpha coefficient is the most extensively used coefficient to assess the internal consistency metric, which can measure the different characteristics of the same construct [49,60]. The employment of many factors or items to gain information about a specific construct would ensure that the dataset is more reliable [65]. In the present study, all items were highly related for each construct as the Cronbach alpha values were within acceptable and outstanding ranges. It could be concluded that the attributes of similar factors in our questionnaire measure the same phenomenon. However, Cronbach’s alpha would indirectly indicate the degree to which a group of elements only assesses a particular unidimensional latent construct. Thus, it is important to note that Cronbach’s alpha should be employed when different practical areas in the factors are measured within a single construct [65]. This is because different fundamental factorial constructs may return similar values of Cronbach’s alpha [95,96,97]. The current study found that the KAP questionnaire is reliable and accurate in assessing attitudes, practices, and perceptions toward the prevention of food poisoning among school children.

To the best of our knowledge, this questionnaire is specifically developed for school children’s knowledge and attitudes concerning food poisoning and its preventive practices during their mealtime at schools. In addition, six elements of perception are also designed according to the Health Belief Model (HBM). The HBM is a comprehensive tool used to explore why an individual is not willing to use screening tests and adopt preventive healthcare recommendations for diseases without clinical manifestations [98,99]. Hence, it is able to evaluate students’ perceptions of threats posed by a health problem (food poisoning), the benefits of avoiding it, and the factors that may influence their decision to react. If a framework based on the HBM is appropriately designed, strategies for behavior change can be implemented [99]. This is supported by a study that demonstrated significantly higher scores in all elements of the HBM on the prevention of brucellosis in the intervention group compared to the control group among Iranian school students. The authors suggested that the HBM is a good model for predicting the prevention of brucellosis in rural students [100].

However, there are several limitations in our study. The present study was conducted among 30 school children as respondents. In terms of the sample size of pilot studies, many pilot studies do not justify the permissible sample size for pilot research. However, Whitehead et al. [101] stated that, for a pilot study, at least 30 respondents are required [101]. In addition, Julious [102] conducted a sample size thumb rule survey about an innovative and supported intervention for individuals with stroke for a pilot study [102]. The author concluded that 12 participants are sufficient to achieve a credible mean and variance [102]. Therefore, because the present study had a small sample size, we were unable to use advanced psychometric testing (e.g., principal component analysis or confirmatory factor analysis) to examine the factor structure of the newly developed questionnaire in the present study. Specifically, such advanced psychometric testing requires a large sample size of over 200 [48,103,104,105]. Moreover, with the small sample size in the present study, the internal consistency could only be examined using Cronbach’s alpha, which has a strong assumption of tau equivalence. Tau equivalence is hard to satisfy, and alternative options such as McDonald’s omega have been proposed [106,107]. However, the small sample size was insufficient for calculating McDonald’s omega. Therefore, future studies with a larger sample are required to re-evaluate the internal consistency of the newly developed questionnaire in the present study. In addition, the present study did not have an external criterion measure; therefore, the discriminant validity and convergent validity could not be examined. Additionally, the small sample size could not be tested using factor analysis to examine whether the subscales were divergent from each other. Future studies are needed to further examine the discriminant validity and convergent validity of the newly developed questionnaire.

Our study utilized questions in a closed-ended format, which did not allow the respondents to express or elaborate their own responses. However, since this is the first pilot study conducted among school children, this format made it easier for them to respond to or engage with the survey. Lastly, the current study was limited to Tangkak secondary school students, and the test–retest reliability should be assessed to ensure that the measurements obtained in one session are representative and constant over time.

## 5. Conclusions

This study highlights the feasibility of conducting a survey using a valid and reliable KAP questionnaire as an appropriate tool for assessing the knowledge, attitudes, practices, and perceptions toward the prevention of food poisoning among secondary school students. This questionnaire covers the most important constructs of the HBM for assessing students’ KAP of food poisoning. It is hoped that this tool will be able to identify influencing factors that may change students’ behavior toward safe eating habits.

## Figures and Tables

**Table 1:** Participants’ sociodemographic characteristics (N = 15 in the pre-test group phase 1 and 30 in the pilot study group phase 2).

Characteristics	N (%) for Pretest	N (%) for Pilot Study
Sex		
Male	6 (40.0)	9 (30.0)
Female	9 (60.0)	21 (70.0)
Age (in years)		
13	3 (20.0)	6 (20.0)
14	3 (20.0)	6 (20.0)
15	3 (20.0)	6 (20.0)
16	3 (20.0)	6 (20.0)
17	3 (20.0)	6 (20.0)
Ethnicity		
Malay	7 (46.7)	18 (60.0)
Indian	5 (33.3)	6 (20.0)
Chinese	3 (20.0)	6 (20.0)
Household Category *		
B40	8 (53.3)	21 (70.0)
M40	5 (33.3)	9 (30.0)
T20	2 (13.3)	0 (0)

* Note: The household category is based on the median monthly household income of the Malaysian population, which is divided into three categories: the bottom 40% of low-income earners (B40), the middle 40% of average-income earners (M40), and the top 20% of top-income earners (T20), with median monthly incomes of MYR 3000.00, MYR 6275.00, and MYR 13,148.00, respectively [35].

**Table 2:** The difficulty index and discrimination index for each item of the knowledge construct of food poisoning (N = 30).

	Mean Score	Difficulty Index	Discrimination Index	Action
Causes of food poisoning				
Bacteria	1.00	0.80	0.35	Included
Viruses	2.40	0.83	0.15	Amended
Parasites	2.20	0.56	0.10	Amended
Chemicals	1.80	0.90	0.35	Included
Mosquitos	2.60	0.94	0.35	Included
High-risk foods				
Chicken	2.00	0.86	0.20	Included
Meat	2.20	0.94	0.35	Included
Bread	1.60	0.90	0.25	Included
Dried food	2.00	0.86	0.25	Included
Dairy products	1.40	0.94	0.25	Included
Seafood	2.67	0.94	0.15	Amended
Rice	1.80	0.86	0.40	Included
Canned food	1.40	0.94	0.35	Included
Vegetables	2.30	0.90	0.00	Amended
Fruits	2.67	0.86	0.00	Amended
Expired food	2.00	0.94	0.35	Included
Symptoms of food poisoning				
Diarrhea	1.00	0.94	0.15	Included
Vomiting	1.00	0.86	0.10	Amended
Abdominal pain	1.80	0.78	0.35	Included
Dryness of lips	2.60	0.34	0.20	Amended
Feeling tired	2.40	0.46	0.35	Included
Fever	2.60	0.28	0.25	Included
Complications of food poisoning				
Dehydration	2.40	0.94	0.25	Included
Kidney failure	2.60	0.86	0.25	Included
Fatal	2.60	0.94	0.25	Included
Prevention of food poisoning				
Wash your hands after sneezing/coughing	1.00	0.90	0.35	Included
Wash your hands before eating	1.00	0.86	0.20	Amended
Food handlers must have good personal hygiene	1.00	0.94	0.35	Included
Wash fruits before eating	1.00	0.78	0.25	Included
Check (see, taste, and smell) the food before eating	1.00	0.68	0.25	Included
Foods that need to be stored in the refrigerator (4 °C)				
Cut fruits	2.20	0.66	0.35	Included
Cake (with frosting)	1.80	0.88	0.15	Amended
Cooked food should be kept at a temperature of 65 °C and above	2.20	0.90	0.35	Included
Cooked food can be stored at room temperature for more than 4 h	2.20	0.92	0.35	Included

**Table 3:** Item analysis of attitude toward prevention of food poisoning (N = 30).

	Range Inter-Item Correlation	Corrected Item—Total Correlation	Cronbach’s Alpha If Item Deleted	Action
I will ensure the food that I buy is clean and safe.	0.426–0.476	0.786	0.933	Included
I will ensure the canteen that I visit is clean.	0.476–0.537	0.763	0.935	Included
I will always make sure to wash hands with soap before eating.	0.426–0.537	0.717	0.937	Included
I do not worry if there are pests (rodents/flies/cockroaches) in the canteen.	0.426–0.476	0.786	0.933	Included
I will always make sure to check the expiry date of food before I buy.	0.426–0.476	0.786	0.933	Included
I do mind if I see food handlers wearing stained aprons.	0.476–0.537	0.763	0.935	Included
I am not worried if I have symptoms of food poisoning.	0.426–0.537	0.717	0.937	Included
I will ensure the teachers are informed if I develop symptoms of food poisoning.	0.426–0.476	0.786	0.933	Included
I will ensure the complaint is lodged to the teacher if there are unhygienic activities in the canteen.	0.476–0.537	0.763	0.935	Included
I will always make sure to check the physical condition (unusual foaming/discoloration) of food before buying.	0.426–0.537	0.717	0.937	Included

**Table 4:** Item analysis of practice toward prevention of food poisoning.

	Range Inter-Item Correlation	Corrected Item—Total Correlation	Cronbach’s Alpha if Item Deleted	Action
I buy food that is clean and safe.	0.272–0.408	0.545	0.846	Included
I do not eat at the canteen if it is dirty.	0.167–0.522	0.396	0.855	Included
I wash my hands until clean before eating.	0.067–0.816	0.669	0.831	Included
I inform teachers if there are pests (such as rodents/flies/cockroaches) in the canteen.	0.167–0.522	0.396	0.855	Included
I check the expiry date of food before buying.	0.136–0.816	0.538	0.843	Included
I smell food first to make sure it is not spoiled.	0.067–0.605	0.413	0.854	Included
I only eat properly cooked meat.	0.167–0.816	0.793	0.820	Included
I inform teachers if I experience food poisoning symptoms.	0.067–0.522	0.456	0.850	Included
I lodge a complaint to my teacher if there are unhygienic activities in the canteen.	0.167–0.816	0.793	0.820	Included
I check the physical condition (unusual foaming/discoloration) of food before buying.	0.067–0.816	0.669	0.831	Included

**Table 5:** Item analysis of perception toward prevention of food poisoning.

	Range Inter-Item Correlation	Corrected Item—Total Correlation	Cronbach’s Alpha If Item Deleted	Action
Health-seeking behavior				
Before eating my meal, I will first check the physical condition (unusual foaming/discoloration) of food. (HSB1)	0.250–0.913	0.396	0.756	Included
Before eating a packed food, I will first check the expiry date. (HSB2)	0.250–0.612	0.824	0.724	Included
I exercise caution if the canteen and activity of food handler looks unhygienic. (HSB3)	0.250–0.896	0.714	0.729	Included
Perceived susceptibility				
The chance of me developing food poisoning is less. (PSus1)	0.140–0.910	0.767	0.858	Included
There is a high possibility that I will eat contaminated food which can cause food poisoning. (PSus2)	0.418–0.767	0.824	0.724	Included
I feel that I could be vulnerable to food-borne pathogens from contaminated food. (PSus3)	0.250–0.767	0.714	0.729	Included
Perceived severity				
Food poisoning can be life-threatening. (Pse1)	0.140–0.873	0.722	0.726	Included
Very few people need to see a doctor because of a food-borne illness. (Pse2)	0.299–0.873	0.830	0.726	Included
Consuming unsafe food can make people fall sick. (Pse3)	0.234–0.612	0.105	0.779	Amended
Perceived benefits				
Checking the physical condition (foaming/discoloration) of food is effective in preventing food poisoning. (Pbe1)	0.250–0.612	0.824	0.724	Included
Exercising care regarding the hygienic activity of food handlers before buying my meal is effective in preventing food poisoning. (Pbe2)	0.167–0.913	0.421	0.759	Included
Exercising care regarding the environment of the canteen before buying my meal is effective in preventing food poisoning. (Pbe3)	0.167–0.764	0.514	0.755	Included
Perceived barriers				
Getting treatment for food poisoning symptoms in a clinic or hospital is time consuming. (Pba1)	0.140–0.873	0.815	0.808	Included
Informing teachers about unhygienic activity in the canteen would require more effort which is inconvenient. (Pba2)	0.250–0.612	0.046	0.773	Amended
Choosing safe food requires more effort which is inconvenient. (Pba3)	0.167–0.963	0.500	0.796	Included
Cues to action				
My teacher gives a talk about food poisoning. (Cta1)	0.218–0.963	0.155	0.773	Amended
My school distributes pamphlets about food poisoning. (Cta2)	0.250–0.802	0.453	0.753	Included
My teacher constantly reminds me to practice hygienic activity. (Cta3)	0.102–0.839	0.165	0.780	Amended
Self-efficacy				
I can recognize food poisoning symptoms. (Se1)	0.134–0.896	0.733	0.733	Included
I am confident of recognizing unhygienic activities by the food handlers. (Se2)	0.250–0.875	0.571	0.744	Included
I am confident of reporting to the teacher if I come across an unhygienic environment in the canteen. (Se3)	0.375–0.875	0.510	0.748	Included

## Data Availability

The dataset that supports the findings of this study is not openly available but can be obtained from the corresponding author upon reasonable request.
